# Production of FeCl_3_-Treated Amine Functional Polymer Gel for Enhanced Removal of Methyl Orange and Congo Red Anionic Dyes

**DOI:** 10.3390/polym18151838

**Published:** 2026-07-27

**Authors:** Şeyda Getir, Atakan Toprak, Baki Hazer

**Affiliations:** 1 Department of Chemistry and Chemical Process Technology, Zonguldak Bülent Ecevit University, 67900 Zonguldak, Turkey; 2 Department of Nano Technology Engineering, Zonguldak Bülent Ecevit University, 67100 Zonguldak, Turkey; baki.hazer@kapadokya.edu.tr; 3 Department of Chemistry, Zonguldak Bülent Ecevit University, 67100 Zonguldak, Turkey; 4 Department of Aircraft Airframe Engine Maintenance, Kapadokya University, 50420 Nevşehir, Turkey

**Keywords:** FeCl_3_ treatment, polymer gel, anionic dye, adsorption thermodynamics, adsorption kinetics

## Abstract

Synthetic dyes such as Methyl Orange (MO) and Congo Red (CR) are recalcitrant, toxic, mutagenic and resistant to conventional biodegradation, and their industrial discharge continues to contaminate aqueous ecosystems worldwide. This study reports the synthesis and characterization of a poly(MMA-co-2-AEMA) copolymer gel (Fe-Copolymergel-NH_2_) treated with FeCl_3_. It evaluates its adsorption performance from aqueous solution against the anionic azo dyes MO and CR. Extensive characterization using N_2_ adsorption–desorption at 77 K, FTIR-ATR, XPS, SEM, and TEM revealed that Fe modification significantly improved the material’s textural properties, increasing its specific surface area from 8.11 m^2^/g to 12.55 m^2^/g and creating a highly irregular, interconnected, sponge-like morphology. Adsorption experiments showed that Fe-Copolymergel-NH_2_ achieved competitive Langmuir maximum monolayer adsorption capacities of 3201.9 mg/g for CR and 1089.8 mg/g for MO. Kinetic modeling demonstrated that adsorption strictly followed the PSO model, primarily governed by chemisorption via electrostatic attractions and complexation within the internal structure at Fe^3+^ coordination centers. Thermodynamic analysis revealed that the dye removal process was spontaneous and exothermic. Consequently, the successful integration of Fe^3+^ coordination centers resolves the adsorption and structural limitations of pure polymer matrices, positioning Fe-Copolymergel-NH_2_ as a highly promising and efficient adsorbent for the remediation of dye-contaminated industrial wastewater.

## 1. Introduction

The pervasive contamination of aqueous environments by synthetic dyes poses a critical environmental concern, driving the urgent development of highly efficient, structurally robust adsorbent materials for effective wastewater remediation [1]. The widespread discharge of synthetic dyes from industrial activities, particularly in the textile, paper, and food manufacturing sectors, poses significant threats to aquatic ecosystems and human health [2]. These risks are compounded by the dyes’ recalcitrant nature, chemical stability, inherent toxicity, and resistance to conventional biodegradation processes [3,4]. Among these, MO and CR, both anionic azo dyes extensively used in textile industries, are particularly problematic owing to their widespread application and potential to induce severe toxic effects, including skin and eye damage, mutagenicity, carcinogenicity, kidney failure, genetic mutations, cancer, and disruptions to respiratory and reproductive systems in aquatic life and humans [5,6]. Consequently, the effective removal of these dyes from industrial wastewater streams is critical for environmental protection and public health [3,7]. This imperative has catalyzed extensive research into various advanced wastewater treatment technologies, including chemical precipitation, photocatalysis, membrane separation, electrochemical treatment, coagulation-flocculation, ion exchange, biological methods, membrane filtration, photochemical degradation, and anaerobic bioremediation [8,9,10]. Among these techniques, adsorption has emerged as a prominent method owing to its cost-effectiveness, operational simplicity, high efficiency in removing a broad spectrum of pollutants, absence of toxic byproducts, ease of application, straightforward and scalable synthesis of adsorbents, flexibility, and environmental benignity [11]. Polymer-based adsorbents, particularly hydrogels, have garnered considerable interest due to their tunable physicochemical properties, high surface area, exceptional adsorption capacities for various organic pollutants, including anionic dyes like MO and CR, high swelling ratios, and porous microstructures enabling mechanisms such as electrostatic attraction, hydrogen bonding, van der Waals forces, and π–π interactions [12,13,14]. However, current hydrogel adsorbents often suffer from limitations, such as low adsorption capacities, poor mechanical stability, and difficulty in regeneration, which hinder their large-scale practical deployment for wastewater treatment [15]. Consequently, there is an urgent need to develop novel, mechanically robust, and highly efficient polymeric hydrogel systems—such as those utilizing metal-ligand coordination—to overcome these deficiencies and enable sustainable remediation [16,17]. Such modifications facilitate superior adsorption of recalcitrant organic pollutants, including anionic dyes, by introducing specific interactions that extend beyond conventional physisorption. Furthermore, incorporating positively charged moieties within these polymeric networks significantly enhances their affinity and removal efficiency for anionic dyes [18]. Within this landscape, cationic polyelectrolyte hydrogels bearing protonatable –NH_2_ and quaternary-ammonium groups together with related Fe^3+^-coordinated networks have attracted particular interest as high-capacity platforms for anionic azo-dye removal because the electrostatic pairing with the –SO_3_^−^ substituents of MO and CR drives q_0_ values that exceed those of activated carbons and biochars of comparable cost [6,14,15].

When recent studies on dye adsorption of polymer gels are examined, Toprak et al. synthesized an NH_2_-functionalized copolymer gel from vinyl benzyl chloride and 2-aminoethyl methacrylate, followed by FeCl_3_-catalyzed Friedel-Crafts hyper-crosslinking to yield HyPolygel-NH_2_ with superior capacities of 5234 mg/g for CR and 1816 mg/g for MO via electrostatic interactions and hydrogen bonding [4]. Similarly, Zhang et al. fabricated maleic anhydride-acrylamide copolymer-based sodium alginate hydrogels (MAH@AA-P/SA/H) that demonstrated maximum adsorption capacities of 685 mg/g for CR and 653 mg/g for methylene blue, alongside high removal of metal ions such as Cu^2+^ (754 mg/g) and Cr^6+^ (738 mg/g) [19]. Further advancements include the development of trimethylammonium chloride- and methacrylamide-based hydrogels, which achieved impressive methyl orange adsorption capacities of 950 and 994 mg/g, respectively [14]. Furthermore, poly(acrylamide-co-methacryloxyethyltrimethylammonium chloride) hydrogels have demonstrated remarkable adsorption capacities of 840.85 mg/g for CR within 10 min, with the overall process being governed by chemisorption and exhibiting good reusability [3]. In a different approach, Li et al. developed quaternary ammonium hydrogels that achieved over 99% removal efficiency for Direct Red 23, with a maximum adsorption capacity of 1540.19 mg/g, highlighting the potential for highly efficient anionic dye removal through targeted cationic polymer design [20]. These high adsorption capacities and efficiencies underscore the potential of cationic hydrogels as effective adsorbents for anionic dyes. Conversely, cyclodextrin-acrylamide-based adsorbents have demonstrated high efficiency in removing both cationic and anionic dyes, achieving rapid equilibrium times of 15–20 min and significant adsorption capacities for MO [21]. Despite these advancements, many hydrogels still face challenges related to mechanical robustness, reusability, and broad applicability across diverse wastewater matrices. For instance, while xanthan gum-based hydrogels show promise for dye removal, their efficacy is often limited by factors such as ionic strength and pH, necessitating careful optimization for practical applications [22]. These limitations highlight the ongoing need for novel hydrogel architectures that balance enhanced structural integrity and chemical stability with high adsorptive performance across a broader range of environmental conditions.

In this study, we synthesized a mesoporous amine hydrogel, Fe-Copolymergel-NH_2_, by treating a poly(MMA-co-2-AEMA) copolymer gel with FeCl_3_. The textural characterization, surface chemistry, and adsorption behavior of this hydrogel for MO and CR were investigated. The adsorption kinetics (PFO and PSO), equilibrium (Langmuir/Freundlich isotherm), and thermodynamics of copolymergel-NH_2_ and Fe-copolymergel-NH_2_ were systematically studied. It was determined that Fe-copolymergel-NH_2_ is highly effective in CR adsorption.

## 2. Materials and Methods

### 2.1. Materials

2,2′-Azobisisobutyronitrile (AIBN) was purchased from Sigma-Aldrich (St. Louis, MO, USA), and CR≥75.0% was obtained from Acros Organics (Geel, Belgium). FeCl_3_ was supplied by Isolab Chemical (Istanbul, Turkey). MO, 1,2-dichloroethane (DCE), N, N-dimethylformamide (DMF), methyl methacrylate (MMA), and 2-aminoethyl methacrylate (2-AEMA) were purchased from Merck (Darmstadt, Germany). All chemicals were of analytical grade and used as received without further purification.

### 2.2. Preparation of Copolymergel-NH_2_

The NH_2_-functionalized copolymer gel was synthesized via free-radical polymerization [4,23]. In a typical experimental procedure, 5.21 g of DMF, 3.24 g of 2-AEMA, and 7.12 g of MMA were dissolved in a Pyrex tube, and the reaction mixture was prepared by adding 17 mg of AIBN. Prior to the reaction, the mixture was purged with Ar gas to remove dissolved oxygen and prevent potential inhibition of the radical species. Polymerization was then carried out in a temperature-controlled reaction vessel at 80 °C for 2 h. After the reaction was complete, the resulting gel-like product was purified and washed with pure H_2_O to remove unreacted monomers and residual initiator. The purified copolymer gel was then dried at 40 °C, yielding 6.82 g.

### 2.3. Preparation of Fe-Copolymergel-Nh_2_

**Scheme 1:**
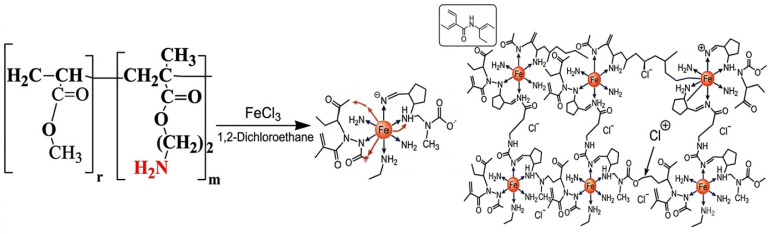
Experimental mechanism diagram of Fe- and Copolymergel-NH_2_.

The Fe-modified polymer hydrogel, designated as Fe-Copolymergel-NH_2_, was synthesized via FeCl_3_-mediated coordination crosslinking following the literature procedure, and the proposed binding geometry is illustrated in Scheme 1 [4]. Initially, the previously synthesized Copolymergel-NH_2_ was placed in a reaction vessel and subjected to swelling in DCE for 2 h. This process was conducted under an argon atmosphere with slow magnetic stirring to ensure the polymer network was sufficiently expanded. Subsequently, the mixture was cooled to approximately 0 °C in an ice bath. 1.52 g of anhydrous FeCl_3_ was then added to the cooled PolygelNH_2_ mixture, and the mixture was stirred for 10 min to achieve a homogeneous dispersion. The reaction temperature was then gradually increased to 80 °C, and the mixture was stirred continuously for 24 h. Following the reaction, the resulting Fe-Copolymergel-NH_2_ was purified by multiple washes with methanol and deionized water, then centrifuged. As a result of this step, Fe-Copolymergel-NH_2_ with improved surface and pore properties was synthesized by removing FeCl_3_ and unreacted substances.

### 2.4. Characterization

The specific surface area (S_BET_) and pore size distributions (PSD) of Copolymergel-NH_2_ and Fe-Copolymergel-NH_2_ were determined through N_2_ adsorption/desorption isotherms using a surface area and pore size analyzer, Quantachrome-Autosorb 1C (Quantachrome, Boynton Beach, FL, USA). The surface morphology and internal structural characteristics were examined via scanning electron microscopy (SEM, FEI QUANTA FEG 450, Graz, Austria) and transmission electron microscopy (TEM, Hitachi HT-7700, Tokyo, Japan), respectively. To evaluate the surface functional groups, Fourier transform infrared spectroscopy (FTIR-ATR, PerkinElmer, Santa Clara, CA, USA) was used. Additionally, changes in the crystalline structure were analyzed using X-ray diffraction (XRD; PANalytical Empyrean, Worcestershire, UK) over a 2θ range of 10–90°. The surface chemical components and electronic states were further characterized by X-ray photoelectron spectroscopy (XPS; Thermo Scientific K-Alpha, East Grinstead, UK) using an Al Kα monochromatic X-ray source.

### 2.5. Adsorption Experiments

Batch adsorption experiments were conducted to evaluate the performance of copolymer gel-NH_2_ and Fe-copolymer gel-NH_2_ adsorbents for the removal of anionic dyes from aqueous solutions. The procedure involved a 50 mL solution volume maintained at a constant agitation speed of 400 rpm, with dye concentrations quantified by UV-Vis spectrophotometry over the 200–800 nm spectral range. Adsorption isotherm and kinetic equilibrium studies were carried out for MO and CR anionic dyes at 100 mg/L in 50 mL using varying dosages of the polymer gel adsorbents (1, 2, 3, 4, and 5 mg). Furthermore, the influence of temperature and thermodynamic parameters was evaluated over the range of 20 °C to 40 °C. The equilibrium adsorption capacities (q_e_) of the dyes were calculated according to the following equation [24]:

(1) $$q_{e}=\frac{(C_{0}-C_{e})\cdot V}{m}$$

### 2.6. Adsorption Kinetics

To elucidate the rate-controlling mechanisms and investigate the temporal behaviour of the adsorption process for Copolymergel-NH_2_ and Fe-Copolymergel-NH_2_, the experimental data were fitted to pseudo-first-order (PFO) and pseudo-second-order (PSO) kinetic models. The PFO kinetic model implies that the adsorption process is governed by physical interactions, including H_2_ bonding, π-π stacking, and van der Waals forces, established between the functional groups of the adsorbent matrix and the aromatic structure of the dye molecule. The following equation expresses the linear form of the PFO model [24,25]:

ln(q_e_ - q_t_) = ln q_e_ - k_1_ t (2)

where q_e_ and q_t_ (mg/g) represent the adsorption capacities at equilibrium and at time t (min), respectively, and k_1_ (min^−1^) denotes the PFO rate constant.

The PSO kinetic model, which posits that the rate-limiting step involves chemisorption through electron sharing or exchange between the adsorbent and the dye molecules, is expressed as [26]:

(3) $$\frac{t}{q_{t}}=\frac{1}{k_{2}q_{e}^{2}}+\frac{t}{q_{e}}$$

in this expression, k_2_ (g mg^−1^ min^−1^) is the PSO rate constant.

### 2.7. Adsorption Isotherms Models

To investigate the equilibrium relationship between the target anionic dyes and the synthesized adsorbents, the experimental data were analyzed using the Langmuir and the Freundlich isotherm models. These models are essential for characterizing the interaction between the dye molecules and the Fe-Copolymergel-NH_2_ surface. The Langmuir model, which assumes that adsorption occurs on a homogeneous surface through monolayer coverage with no interaction between adsorbed molecules, is expressed in its linear form as follows [4]:

(4) $$\frac{C_{e}}{q_{e}}=\frac{1}{K_{L}q_{0}}+\frac{C_{e}}{q_{0}}$$

In this equation, C_e_ (mg/L) is the equilibrium concentration of the dye, q_e_ (mg/g) is the amount of dye adsorbed at equilibrium, q_0_ (mg/g) represents the maximum monolayer adsorption capacity, and K_L_ (L/mg) is the Langmuir constant related to the energy of adsorption [27].

The Freundlich isotherm model is employed to describe multilayer adsorption on heterogeneous surfaces with sites of varying affinities. The linear mathematical representation of the Freundlich model is given by [28]:

(5) $$\ln q_{e}=\ln K_{F}+\frac{1}{n}\ln C_{e}$$

where K_F_ (mg/g) is the Freundlich constant representing the adsorption capacity and 1/n is the heterogeneity factor. These parameters, derived from the intercept and slope of the linear regression, provide a comprehensive understanding of the maximum adsorption potential and the interaction favorability between the adsorbent’s surface and anions [24,29].

### 2.8. Adsorption Thermodynamics

To characterize the energetic changes associated with the adsorption process, the thermodynamic parameters, specifically the Gibbs free energy change (ΔG^0^), enthalpy change (ΔH^0^), and entropy change (ΔS^0^), were determined using the van’t Hoff equation [30]. The distribution coefficient (K_d_) employed in the thermodynamic analysis was calculated as:

(6) $$K_{d}=\frac{q_{e}}{C_{e}}$$

where is the equilibrium adsorption capacity, and is the equilibrium dye concentration. The values of ΔH^0^ and ΔS^0^ were obtained from the slope and intercept of the van’t Hoff plot of ln K_d_ versus 1/T, according to:

(7) $$\ln K_{d}=\frac{\Delta S^{0}}{R}-\frac{\Delta H^{0}}{RT}$$

Negative values of ΔG^0^ indicate the spontaneous nature of the adsorption process. At the same time, the signs of ΔH^0^ and ΔS^0^ provide insight into the endothermic or exothermic nature and the degree of disorder at the solid–liquid interface [31]. The standard Gibbs free energy change was computed using the relation:

(8) $$\Delta G^{0}=-RT\ln K_{d}$$

where a decrease in these values with increasing temperature indicates a greater favorability of the adsorption process under thermal influence. The calculated ΔH^0^ and ΔS^0^ values, obtained from the slope and intercept of the van’t Hoff plot, indicate whether the mechanism is dominated by physisorption or chemisorption [32]. Spontaneity was further evaluated through the fundamental Gibbs–Helmholtz relation:

(9) $$\Delta G^{0}=\Delta H^{0}-T\Delta S^{0}$$

### 2.9. Water Swelling Percentage

The swelling behavior of the synthesized copolymer gel was evaluated by immersing a known mass of the dried sample in deionized water until equilibrium swelling was attained. The swollen hydrogel was then removed, carefully blotted with filter paper to eliminate excess surface moisture, and weighed to determine the equilibrium water content. The swelling percentage (S_w_ %) was subsequently calculated using the formula:

(10) $$S_{w}(\%)=\frac{W_{s}-W_{d}}{W_{d}}\times 100$$

where W_s_ and W_d_ represent the weight of the swollen hydrogel at equilibrium and the weight of the dried polymer sample, respectively.

## 3. Results and Discussion

### 3.1. Surface Area and Pore Volume Characterization of Fe-Copolymergel-Nh_2_

**Figure 1:**
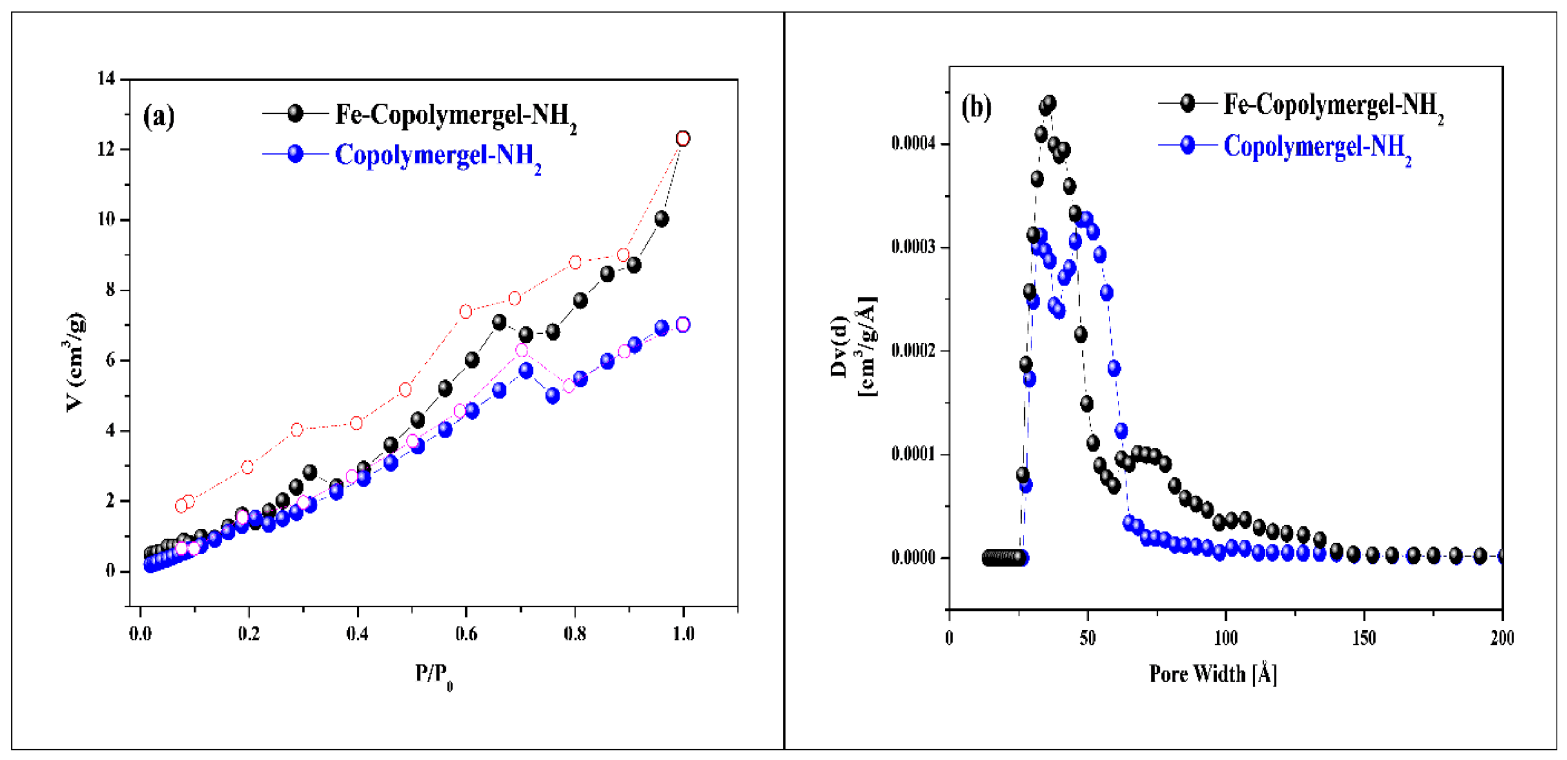
(**a**) N_2_ adsorption/desorption isotherms and (**b**) NLDFT pore size distribution of Fe- and Copolymergel-NH_2_.

The textural properties of Copolymergel-NH_2_ and Fe-Copolymergel-NH_2_ were evaluated through N_2_ adsorption–desorption isotherms and Non-Local Density Functional Theory (NLDFT) pore size distributions, as illustrated in Figure 1. Figure 1a displays the N_2_ adsorption–desorption isotherms for both materials. According to the IUPAC classification, both samples exhibit Type IV isotherms with a distinct H3-type hysteresis loop occurring at relative pressures (P/P_0_) between 0.4 and 1.0 [33,34]. This behaviour is characteristic of mesoporous materials with slit-shaped pores. Notably, the adsorption volume of Fe-Copolymergel-NH_2_ is significantly higher than that of the pristine Copolymergel-NH_2_ across the entire pressure range. The increased N_2_ uptake at high relative pressures (P/P_0_ > 0.8) suggests that the FeCl_3_ treatment and subsequent crosslinking process enhanced the total pore volume and specific surface area, likely due to structural rearrangement or the formation of new coordination sites within the polymer matrix [4].

The pore-size distribution curves, calculated using the NLDFT method, are shown in Figure 1b. Both materials exhibit a predominant pore diameter of 20–60 Å, confirming the mesoporous nature of the copolymer gels. For Fe-Copolymergel-NH_2_, the peak intensity is markedly higher compared to Copolymergel-NH_2_, indicating a higher density of mesopores within this size regime. This increase in porosity and available surface area is expected to facilitate the diffusion and sequestering of bulky anionic dye molecules, such as MO and CR, thereby enhancing the overall adsorption capacity [35].

The textural properties of Copolymergel-NH_2_ and Fe-Copolymergel-NH_2_ were calculated through N_2_ adsorption–desorption isotherms and NLDFT pore size distributions at 77 K, as shown in Table 1. The FeCl_3_ treatment of the NH_2_-copolymergel resulted in a significant enhancement of the specific surface area and pore structure. The BET surface area increased from 8.11 m^2^/g for Copolymergel-NH_2_ to 12.55 m^2^/g for Fe-Copolymergel-NH_2_, representing an increase of approximately 55%. This improvement suggests that incorporating Fe^3+^ ions and their subsequent coordination with amine groups within the polymer matrix likely prevented the collapse of the polymer network or induced structural rearrangements that exposed more active surface sites.

Analysis of the pore volume data reveals a clear shift toward higher porosity following the iron treatment. The total pore volume (V_t_) increased from 0.0096 to 0.0135 cm^3^/g, primarily driven by the expansion of the mesoporous volume (V_meso_), which rose from 0.0109 to 0.0125 cm^3^/g. Conversely, the microporous volume (V_DRmicro_) showed a slight decrease from 0.0013 to 0.0010 cm^3^/g, suggesting that some micropores may have been merged into larger mesopores or partially obstructed by the iron coordination complexes. The high mesoporous-to-micropore volume ratio minimizes mass-transfer resistance for these large dye molecules, allowing efficient diffusion into the internal active sites of the Fe-Copolymergel-NH_2_ adsorbent.

### 3.2. FTIR and XPS Analysis

**Figure 2:**
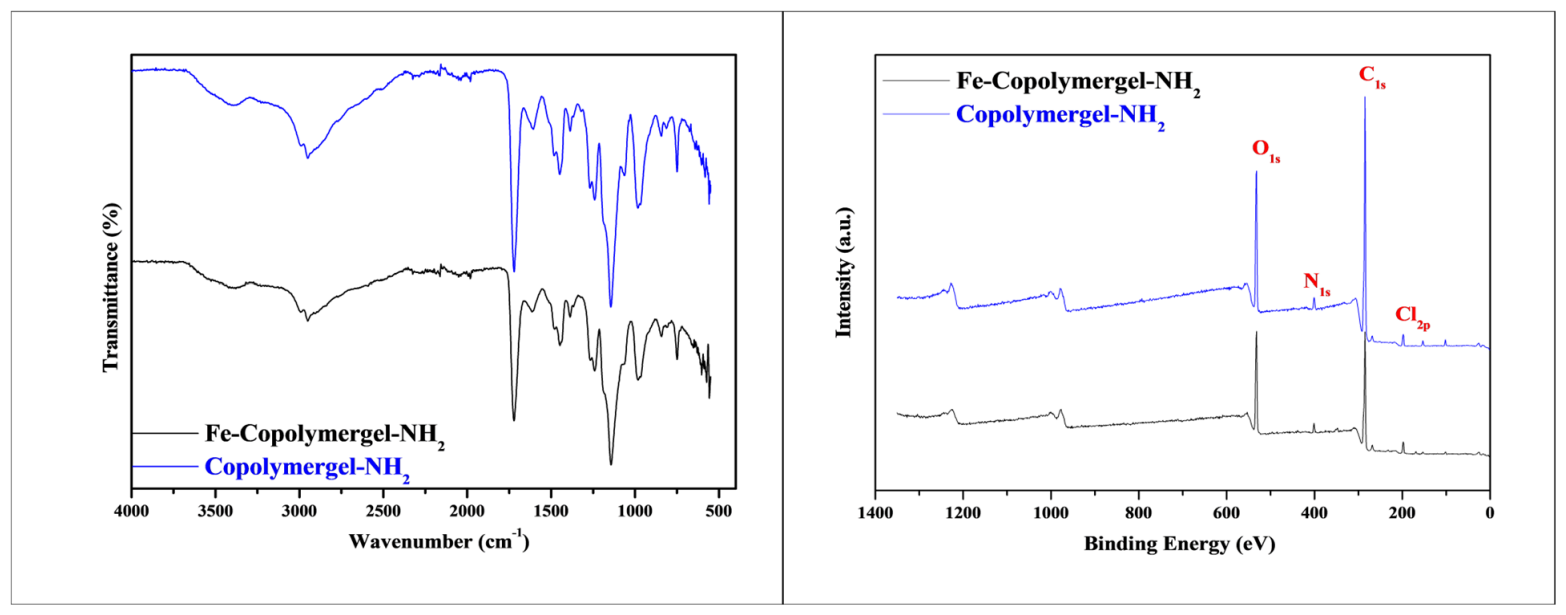
The FTIR spectra of Fe- and Copolymergel-NH_2_.

As shown in Figure 2, the chemical structures and functional groups of pure Copolymer Gel-NH_2_ and modified Fe-Copolymer Gel-NH_2_ were investigated using FTIR-ATR spectroscopy and XPS. The spectrum of the copolymergel-NH_2_ exhibits the characteristic peaks of the poly(MMA-co-2-AEMA) structure. A broad absorption band at approximately 3400–3500 cm^−1^ is assigned to the N–H stretching vibrations of the primary amine groups [4]. The peaks observed at 2950 cm^−1^ and 2870 cm^−1^ correspond to the C–H asymmetric and symmetric stretching of the CH_2_ and CH_3_ groups in the polymer chain. The sharp, intense peak at 1723 cm^−1^ is a defining feature of ester groups, corresponding to the C=O stretching vibration. Additionally, it shows a strong band at 1145 cm^−1^ attributed to the C–O–C stretching vibration of the ester bond [36,37]. Following the treatment with FeCl_3_, the spectrum of Fe-Copolymergel-NH_2_ retains the primary features, indicating structural stability. However, changes occur in the amine and carbonyl regions. The shift and broadening of the N–H/O–H band around 3400 cm^−1^ suggest the participation of amine lone pairs in coordination with Fe^3+^ ions [38]. Both samples exhibit prominent peaks at C 1s (~285 eV) and O 1s (~532 eV), originating from the PMMA and 2-AEMA monomer units, respectively. The presence of a distinct N 1s peak (~400 eV) in both spectra confirms the successful copolymerization of the amino-functionalized monomer into the gel matrix.

### 3.3. SEM and TEM Analysis

**Figure 3:**
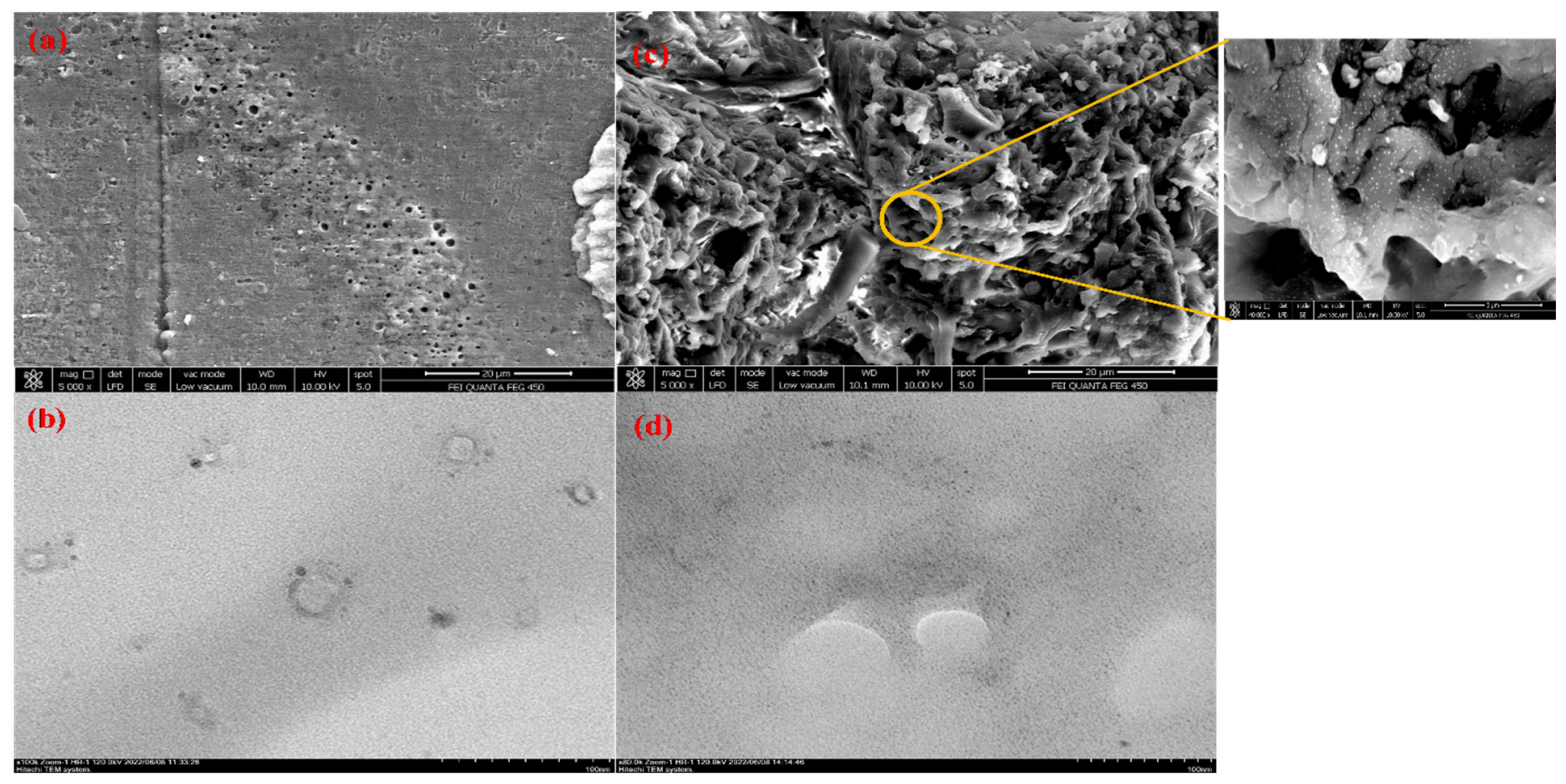
SEM (**a**,**b**), TEM images (**c**,**d**) of Fe- and Copolymergel-NH_2_.

The surface morphology and internal structural properties of the synthesized materials were investigated using SEM and TEM to explore the effect of iron modification on the polymer framework. Figure 3 illustrates the significant morphological transition between the pristine Copolymergel-NH_2_ and the modified Fe-Copolymergel-NH_2_. As shown in the SEM micrograph in Figure 3a, the surface of the pristine Copolymergel-NH_2_ is relatively smooth and continuous, exhibiting only minor surface irregularities and a few visible micropores. This relatively uniform surface suggests a stable but less accessible polymer matrix. In stark contrast, the SEM image of Fe-Copolymergel-NH_2_ (Figure 3c) reveals a dramatic shift toward a highly irregular, rough, and complex morphology. The surface appears highly fragmented, with a sponge-like structure characterized by deep voids and a high density of interconnected pores. Furthermore, in the magnified view of a section of Figure 3c, a regular distribution of iron compounds is identified. This open, rough surface is highly advantageous for wastewater treatment applications, as it provides a larger active area and minimizes mass-transfer resistance for large anionic dye molecules.

The TEM image of Copolymergel-NH_2_ (Figure 3b) shows a relatively low-density internal network with a light, uniform contrast, indicating a standard polymer gel matrix without heavy atom clusters. However, the TEM image of Fe-Copolymergel-NH_2_ (Figure 3d) exhibits a more complex internal density. The appearance of dark, speckled regions and increased contrast across the matrix signifies the successful incorporation of Fe^3+^ species and the formation of a more rigid, dense network.

The successful incorporation of Fe species and the associated structural rearrangement of the polymer matrix were quantitatively confirmed through XPS and SEM-EDS analyses, and the elemental weight percentages are summarized in Tables S1 and S2, respectively. A consistent decrease in C content was observed across both techniques following the FeCl_3_ treatment, with XPS recording a decline from 67.30% to 56.22% and SEM-EDS recording a reduction from 58.32% to 56.22%. This decrease results not from the degradation of the organic skeleton, but rather from the quantitative incorporation of higher-mass Fe and Cl species into the polymer matrix. The appearance of a 4.42 wt.% signal directly confirms Fe loading. Fe signal in SEM-EDS-absent in the pristine sample and indirectly corroborated by the substantial increase in Cl content, which rose from 3.74% to 5.65% in XPS and from 2.19% to 2.88% in SEM-EDS. The redistribution of N (increase from 2.90% to 3.38% in XPS) and O (increase from 22.50% to 27.52% in XPS) further supports the metal-ligand coordination mechanism; it can be said that amine functional units move towards the surface and coordinate with Fe^3+^ ions.

The equilibrium water S_w_ of the pristine Copolymergel-NH_2_ was determined to be approximately 1551.6%, whereas the FeCl_3_-modified Fe-Copolymergel-NH_2_ exhibited a substantially higher swelling capacity of 2104.5% (Figure S1). This increase in hydrogel hydrophilicity is attributed to modifications in the polymer network, where the introduction of Fe-coordinated centers creates additional space and enhances the availability of polar active sites for interaction with water molecules [18].

### 3.4. Adsorption Studies

**Figure 4:**
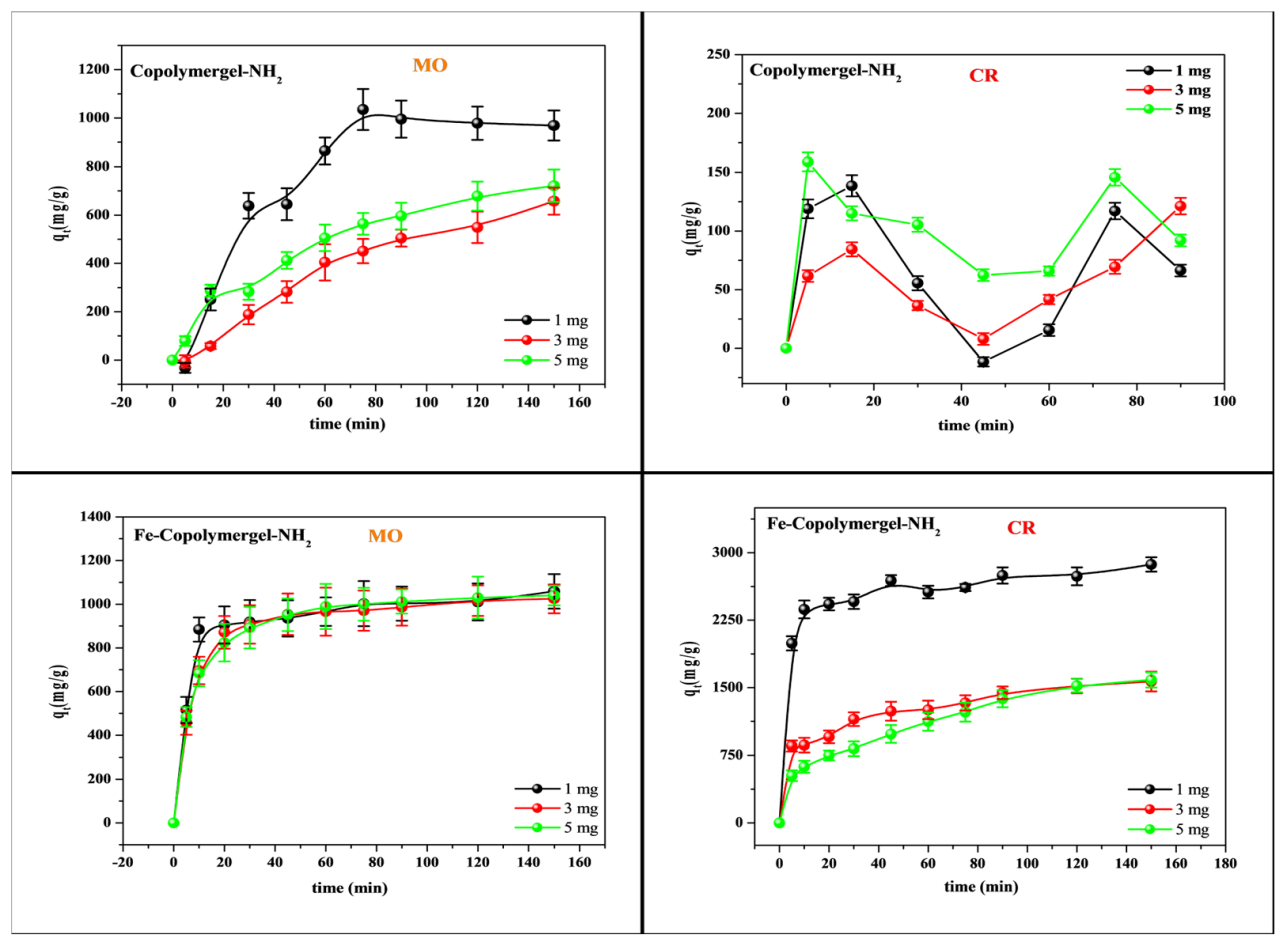
MO and CR adsorption-time graphs of Fe- and Coploymergel-NH_2_ at different doses.

The time variation (t, min.) of the adsorption capacity (q_t_, mg/g) for MO and CR on pure copolymer gel-NH_2_ and Fe-copolymer gel-NH_2_ is given in Figure 4. A significant increase in capacity and stability was observed after the modification process, with the equilibrium qt value for CR adsorption on Fe-copolymer gel-NH_2_ reaching approximately 2800 mg/g at a dosage of 1 mg. In contrast, copolymer gel-NH_2_ exhibited significantly lower q_t_ with considerable unstable fluctuations. The kinetic curves showed a rapid initial uptake phase, during which 80–90% of the total adsorption occurred within the first 20 min, followed by a gradual approach to an equilibrium plateau between 120 and 180 min; this suggests film diffusion control and intraparticle diffusion within the porous network. Importantly, while pure Copolymergel-NH_2_ exhibited irregular q_t_-t due to its smooth, non-porous morphology and insufficient active site density, Fe-Copolymergel-NH_2_ formed smooth curves thanks to its highly porous, structurally robust matrix produced by FeCl_3_-mediated coordination crosslinking.

**Figure 5:**
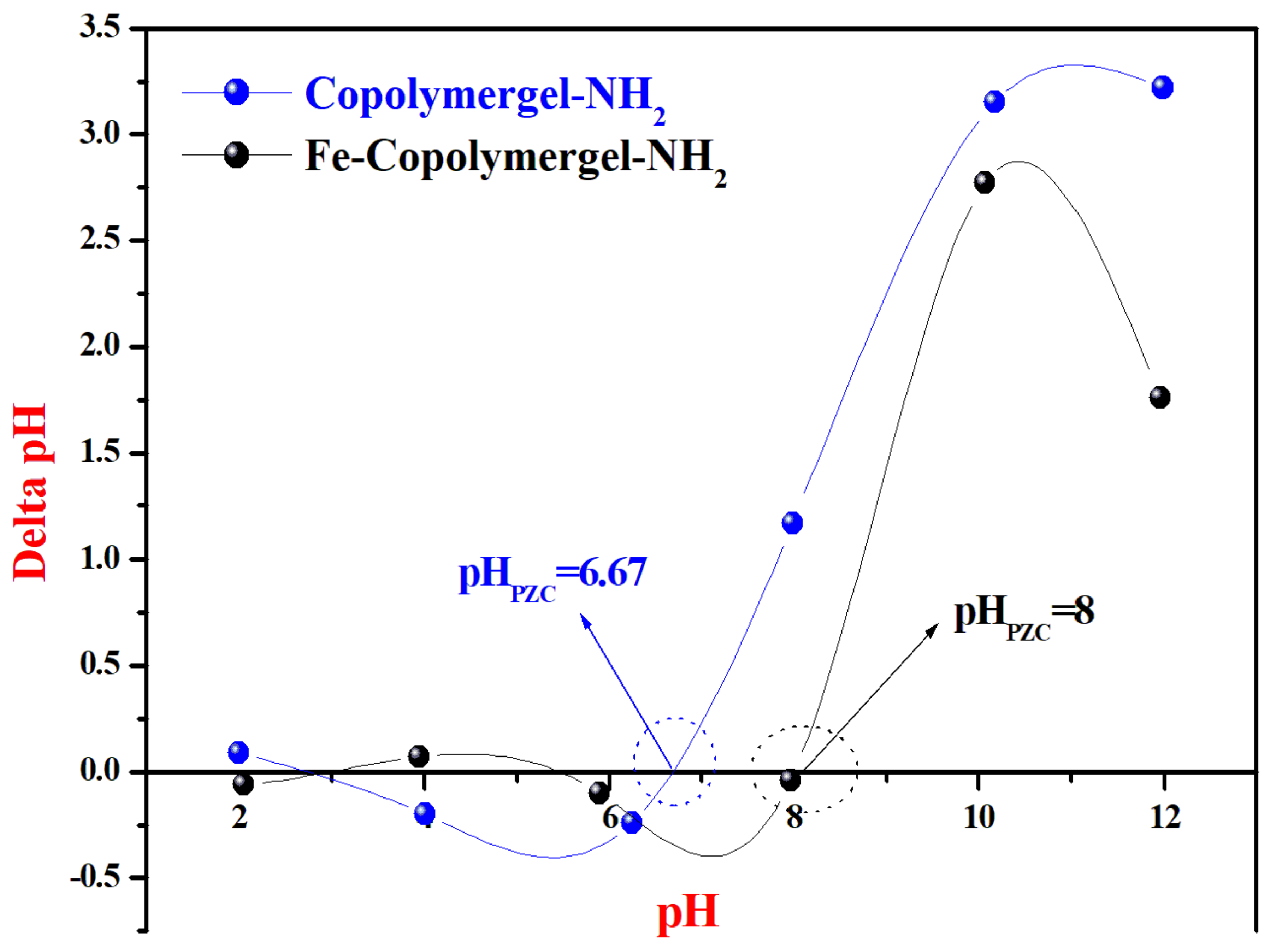
Determination of pH_PZC_ for Fe- and Copolymergel-NH_2_.

In Figure 5, the pH zero charge point (pH_PZC_), determined using the pH drift method, corresponds to the pH at which the net electrical charge on the adsorbent surface drops to zero, thereby defining the electrostatic nature of the interface under aqueous conditions [25]. The Copolymergel-NH_2_ exhibits a pH_PZC_ value of 6.67. In contrast, the FeCl_3_ treatment elevates this parameter to 8.0 in the resultant Fe-Copolymergel-NH_2_, reflecting an increase of approximately 1.33 pH units driven by the redistribution of surface functional groups and the modulation of protonation equilibria following iron coordination. This pH_PZC_ value fundamentally determines the surface ionization state: below this threshold, protonation of surface functionalities generates a net positive charge, whereas above it, deprotonation yields a net negative charge [39]. Consequently, Copolymergel-NH_2_ exhibits a cationic surface only up to pH 6.67. In contrast, the Fe-Copolymergel-NH_2_ counterpart maintains a positive surface potential up to pH 8.0, thereby extending the operational window favorable for electrostatic interactions with target solutes.

### 3.5. Kinetic Studies of Adsorption Processes

**Figure 6:**
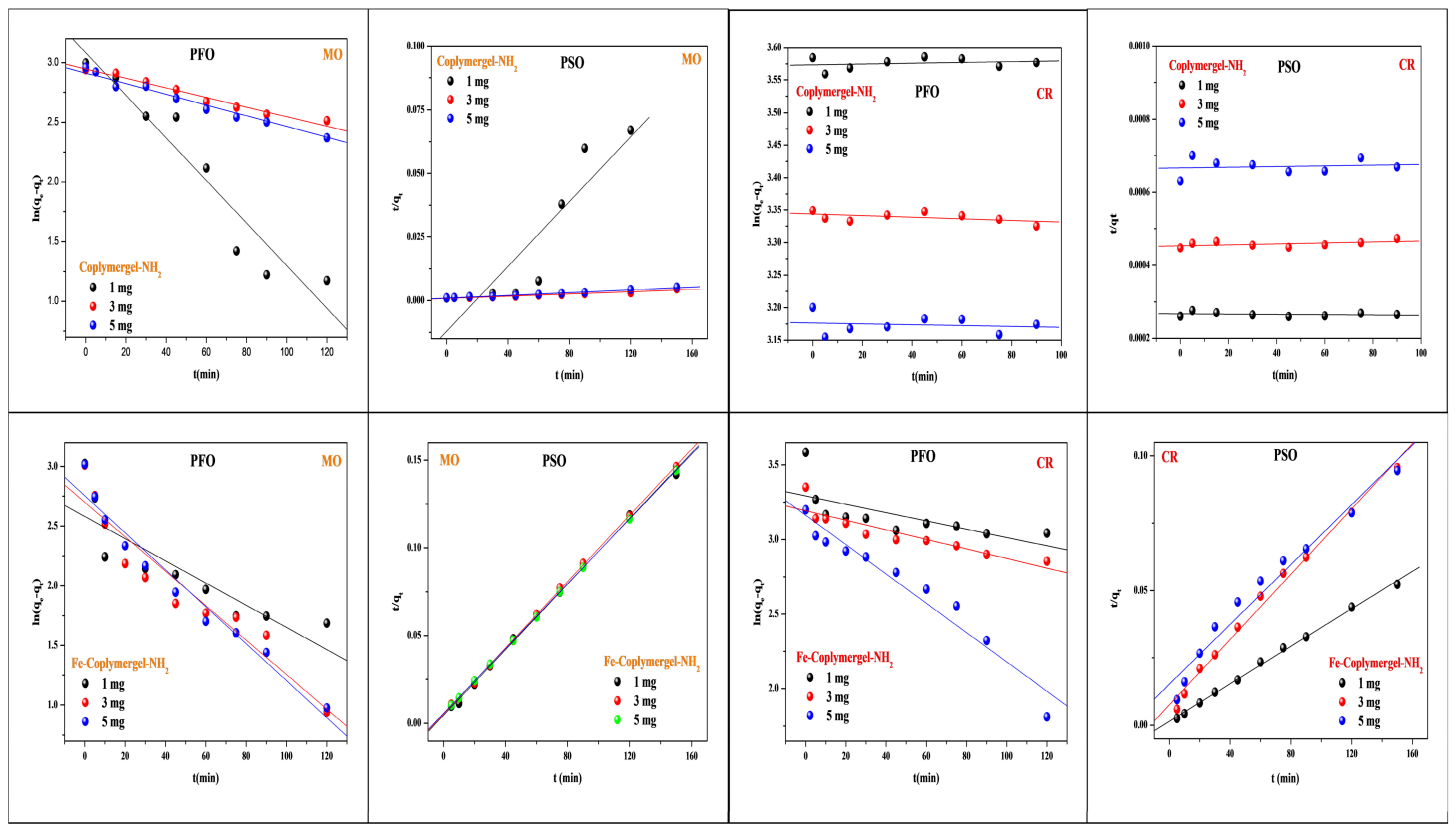
PFO and PSO kinetic models of MO and CR adsorption onto Fe- and Copolymergel-NH_2_ at different doses.

The PFO and PSO kinetic modelling results are summarized in Table 2 and Figure 6. Because both kinetic models are empirical rate laws, neither is, in isolation, evidence of a particular adsorption mechanism; PSO nonetheless localizes the rate-limiting step at the adsorption sites, while PFO localizes it at film diffusion within the bulk solution. For Fe-Copolymergel-NH_2_, the PSO model provided near-perfect fits (R^2^ = 0.968 for CR and R^2^ = 0.999 for MO) with calculated equilibrium capacities close to experiment (q_e_,exp = 2870 mg g^−1^ vs. q_e_,cal = 2872 mg g^−1^ for CR). Mechanistically, the highly porous and therefore interacting Fe-copolymergel-NH_2_ reflects synergistic interactions, including electrostatic attraction between protonated –NH_3_^+^ sites and anionic dye –SO_3_^−^ groups, and inner sphere complexation occurring via added Fe^3+^ centers [40].

In contrast, the low-porosity Copolymergel-NH_2_ exhibited nonphysical compatibility, confirming the structural inadequacy of the unmodified matrix for effective dye uptake. The relatively low CR uptake of pure Copolymergel-NH_2_, despite a certain level of MO adsorption was due to the inability of CR molecules to penetrate the inner parts of the gel because of their larger molecular structure compared to MO, due to its low porosity, and the low level of interaction being solely due to surface interaction. The kinetic graphs in Figure 6 show well-defined linearity for the PSO model and a pronounced curvature for the PFO model, reinforcing that Fe-functionalization successfully establishes a robust chemisorption-driven mechanism.

### 3.6. Adsorption Isotherms

**Figure 7:**
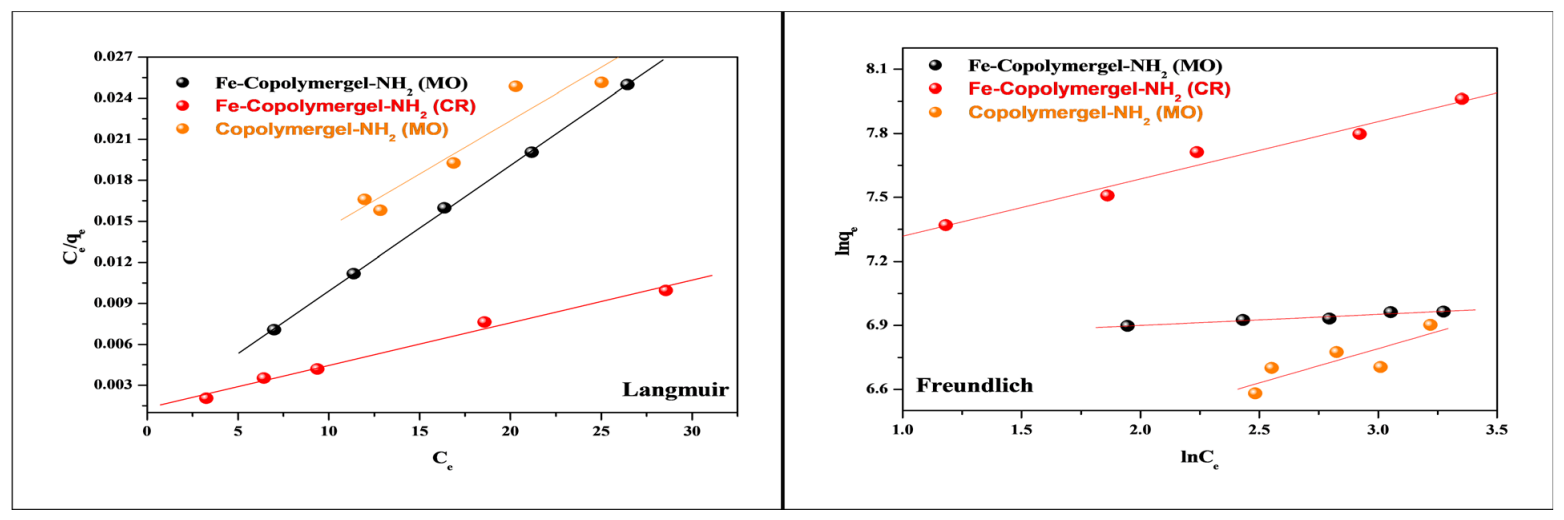
Langmuir and Freundlich isotherms for Fe- and Copolymergel-NH_2_ adsorption of MB and MO at different doses.

The Langmuir and the Freundlich isotherm plots for CR and MO are presented in Figure 7, and the parameters derived from these plots are summarized in Table 3. The higher R^2^ observed for the Langmuir model, particularly for the Fe-modified adsorbent, suggests that the adsorption process is primarily governed by monolayer coverage on a structurally homogeneous surface with a uniform distribution of active sites. Furthermore, the high n values obtained from the Freundlich model indicate strong, favorable adsorption intensity, confirming the energetic heterogeneity of the surface sites facilitated by the Fe^3+^ modifications [41]. This alignment with the Langmuir isotherm underscores the prevalence of localized chemical interactions where dye molecules occupy specific, equivalent coordination centers. These results reflect a monolayer-type chemisorption process in which the saturation of these high-affinity Fe-based sites effectively restricts multilayer deposition. The findings demonstrate that the Fe-functionalized gel possesses one of the highest reported monolayer capacities for CR, with q_0_ reaching 3201.9 mg/g alongside K_L_ of 0.23 L/mg, and similarly elevated Langmuir parameters for MO. In contrast, the pristine Copolymergel-NH_2_ yielded only a moderate MO fit and failed to converge for CR, confirming the indispensable role of Fe^3+^ in establishing well-defined coordination sites. Parallel examination of the Freundlich constants corroborates this conclusion: K_F_ rose from 338.9 for the unmodified matrix to 894.6 for Fe-Copolymergel-NH_2_ toward MO, and from a non-convergent value for CR to K_F_ = 1150.8 with n = 3.72 for the dye on the Fe-modified gel, where n > 1 throughout signals spontaneous and energetically favorable sorption across both substrates [25]. Comparable trends in the energetic favorability of the adsorption process, as indicated by these intensity parameters, align with findings observed in similar metal-doped porous adsorbents such as CoMgAl-LDH and α-Mn_2_O_3_ architectures, where Fe^3+^- or transition-metal-mediated coordinative anchoring dictates the partitioning of anionic contaminants [42,43]. Moreover, the Fe-Copolymergel-NH_2_ exhibits a higher adsorption capacity for CR compared to MO, which is attributed to the larger molecular size of the CR molecule in conjunction with the high porosity of the Fe-Copolymergel-NH_2_ matrix [44]. As summarized in Table 4, Copolymergel-NH_2_ and Fe-Copolymergel-NH_2_ exhibit superior CR and MO adsorption capacities compared to many previously reported adsorbents [44,45,46,47,48,49,50,51,52,53,54,55,56,57,58,59].

### 3.7. Thermodynamic Studies

The thermodynamic parameters for the adsorption of CR and MO onto pristine Copolymergel-NH_2_ and FeCl_3_-mediated Fe-Copolymergel-NH_2_ are compiled in Table 5. These values reveal four distinct thermodynamic behaviors that clarify the impact of the Fe^3+^-amine coordination chemistry. For CR on the pristine Copolymergel-NH_2_, ΔH^0^ = +15.09 kJ mol^−1^ and ΔS^0^ = +58.78 J mol^−1^ K^−1^ together drive an endothermic, entropy-controlled process in which the increasingly negative ΔG^0^ values (−2.14, −2.73, −3.32 and −3.91 kJ mol^−1^ at 293, 303, 313 and 323 K, respectively) reflect the dominant contribution of the positive entropy term as temperature rises, consistent with the release of ordered hydration water molecules from the dye and polymer interface upon adsorption on a structurally under-activated, low-affinity matrix [60]. In contrast, the MO/Copolymergel-NH_2_ system is exothermic and exhibits low entropy (ΔH^0^ = −1.50 kJ mol^−1^, ΔS^0^ = −3.91 J mol^−1^ K^−1^). This confirms the unmodified matrix’s limited capacity for MO adsorption. In contrast, Fe-Copolymergel-NH_2_ exhibits a fundamental thermodynamic shift. CR adsorption becomes strongly exothermic (ΔH^0^ = −34.60$ kJ mol^−1^), with significantly lower ΔG^0^ values (−11.29 to −8.91 kJ mol^−1^). Since |ΔH^0^| >> |TΔS^0^|, the rate-limiting step is governed by valence-strength bond formation; this is the electrostatic attraction between –NH_3_^+^ and –SO_3_^−^ groups, reinforced by the internal structure Fe^3+^ ← :N coordination [61]. A negative ΔS^0^ = −79.50 J/mol · K value indicates that the dye molecules have lost rotational and translational freedom within these coordination structures, consistent with results for similar metal-coordinated hydrogels [60,62]. 

**Figure 8:**
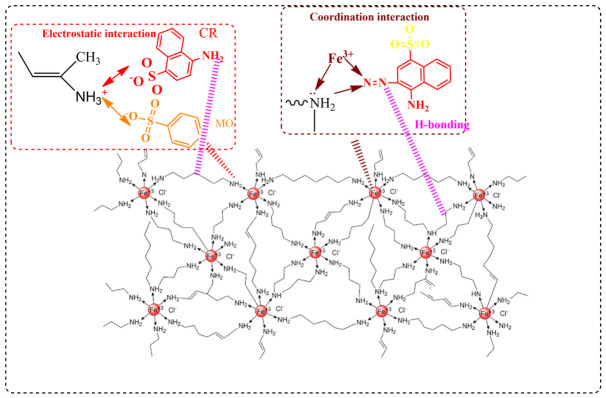
Proposed adsorption mechanism of CR and MO onto Fe-Copolymergel-NH_2_.

MO adsorption on Fe-Copolymergel-NH_2_ also shifts to an exothermic, enthalpy-controlled process (ΔH^0^ = −16.23 kJ mol^−1^, ΔS^0^ = −22.67 J/molK). The systematically more negative ΔG^0^ values demonstrate the effectiveness of the Fe^3+^-induced coordination. The smaller ΔH^0^ value of MO compared to CR can be attributed to its smaller molecular size and lower charge density. Finally, the temperature dependence confirms that room temperature is optimal for adsorption on the Fe-modified gel, as predicted by the Gibbs–Helmholtz relation. Together, these thermodynamic signatures confirm that FeCl_3_-mediated coordination successfully establishes a high-affinity chemisorption mechanism, validating the structural and kinetic evidence presented in earlier sections. As shown in Figure 8, the uptake of CR and MO onto Fe-Copolymer Gel-NH_2_ is governed by three synergistic interactions: electrostatic, coordination, and H-bonding interactions, which operate in parallel at the dative Fe^3+^ ← :NH_2_ coordination nodes placed during the FeCl_3_-catalyzed cross-linking step. In the electrostatic interaction, both CR and MO are dianionic azo dyes bearing -SO_3_^−^ ends, and the protonated -NH_3_^+^ sites formed in the 2-AEMA segments below pH_PZC 7.22 provide a net positive surface charge [4]. Within the Fe-copolymer gel-NH_2_ framework, Fe^3+^ centers doubly chelated by –NH_2_ donors exhibit Fe^3+^ ← :NH_2_ coordination interactions. Unprotonated –NH_2_ groups in the gel structure can donate N–H···N H bonds to dye guests. CR’s higher aromatic ring density and larger molecular footprint compared to MO may allow for stronger π–π stacking and H-bonding with the polymer, supporting the systematically higher uptake of CR compared to MO.

### 3.8. Reusability Studies

The reusability of Fe-Copolymergel-NH_2_ is a crucial parameter for evaluating its industrial viability. To assess this, 3 mg of the adsorbent underwent CR adsorption, followed by regeneration with 0.1 M NaOH (10 min), centrifugation, and drying at 70 °C. Five successive adsorption–desorption cycles were performed, as shown in Figure S2. As illustrated in the bar graph, the material achieved near-quantitative initial CR removal. However, the capacity declined consistently across the five cycles, with removal percentages dropping to roughly 78%, 64%, 51%, and 38% in cycles 2 through 5, respectively. This represents a consistent decrease of approximately 14–17 percentage points per cycle, resulting in ~40% residual activity by the fifth cycle. This disadvantageous performance degradation is attributed to alkali-induced leaching of Fe^3+^–NH_2_ coordination crosslinks formed during FeCl_3_-mediated processing, progressive erosion of the mesoporous skeleton, and irreversible accumulation of CR residues at high-affinity Fe^3+^–amine nodes.

## 4. Conclusions

This study demonstrated that coordination crosslinking of copolymergel-NH_2_, containing primary amine groups, with FeCl_3_ produced a mesoporous adsorbent (Fe-Copolymergel-NH_2_) with high affinity for anionic azo dyes, especially MO and CR. FTIR-ATR, XPS, and SEM-EDS confirmed the characterization of the copolymer gels. FeCl_3_ treatment resulted in a significant increase in the mesoporous properties and surface area (BET, from 8.11 to 12.55 m^2^ g^−1^) of Fe-Copolymergel-NH_2_. These structural changes led to Langmuir maximum capacities Q_0_ = 3201.9 mg g^−1^ and 1089.8 mg g^−1^ for CR and MO in Fe-Copolymergel-NH_2_, respectively. Kinetic modeling revealed that Fe-Copolymergel-NH_2_ exhibits PSO behavior governed by chemisorption of Copolymergel-NH_2_. Thermodynamically, the adsorption of CR and MO is exothermic, facilitating the spontaneous, self-initiated adsorption of Fe-Copolymergel-NH_2_. The reported findings demonstrate that the synthesized Fe-Copolymergel-NH_2_, with its improved pore structure, has significant potential as an effective adsorbent for the removal of anionic dyes such as CR and MO from textile wastewater.

## Figures and Tables

**Table 1:** Pore and surface structural parameters of Fe- and Copolymergel-NH_2_.

Adsorbent	BET (m^2^/g)	V_DRmicro_ (cm^3^/g) (<20 Å)	V_meso_ (cm^3^/g) (20 Å < Vμp)	V_t_ (cm^3^/g) (P/P_0_ = 0.995)
Copolymergel-NH_2_	8.11	0.0013	0.0109	0.0096
Fe-Copolymergel-NH_2_	12.55	0.0010	0.0125	0.0135

**Table 2:** PFO and PSO kinetic model parameters of CR and MO adsorption onto Fe- and Copolymergel-NH_2_.

Adsorbents	Adsorbent Dosage (mg)	q_e_ [exp] (mg/g)	PFO			PSO		
			q_e_ [cal] (mg/g)	k_1_	R^2^	q_e_ [cal] (mg/g)	k_2_ (10^−5^)	R^2^
Copolymergel-NH_2_ (MO)	1	995	134,028	0.011	0.395	258	43.76	0.945
	3	875	311.19	0.012	0.968	403	7.37	0.989
	5	914	257.5	0.010	0.874	353	8.59	0.889
Copolymergel-NH_2_ (CR)	1	219	-	−0.009	0.050	-	5.67	0.053
	3	121	-	0.001	0.261	-	3.77	0.263
	5	92	-	0.001	0.0215	-	1.29	0.019
Fe-Copolymergel-NH_2_ (CR)	1	2870	1958	0.006	0.472	2872	8.54	0.998
	3	2236	1561	0.007	0.809	1649	4.87	0.991
	5	1586	1454	0.023	0.947	1800	2.03	0.968
Fe-Copolymergel-NH_2_ (MO)	1	1059	381	0.021	0.74	1070	19.25	0.999
	3	1025	501	0.033	0.911	1059	17.12	0.999
	5	1040	568	0.036	0.96	1083	15.07	0.999

**Table 3:** Langmuir and Freundlich adsorption isotherm parameters of Fe- and Copolymergel-NH_2_.

Adsorbent	Copolymergel-NH_2_		Fe-Copolymergel-NH_2_	
	MO	CR	MO	CR
Langmuir Model				
*q*_0_ (mg/g)	1281.6	-	1089.8	3201.9
*K*_L_	0.12	-	1.25	0.23
*R* ^2^	0.90	-	0.999	0.989
Freundlich Model				
*K_F_*	338.9	-	894.6	1150.8
*n*	3.11	-	19.25	3.72
*R* ^2^	0.71	-	0.951	0.968

**Table 4:** Adsorption capacities of CR and MO on different adsorbents.

Adsorbents	q_max_ (CR) (mg/g)	q_max_ (MO) (mg/g)	Ref.
CoFe_2_O_4_	162.68	94.46	[44]
PMMA-Na-MT	355.5 mg/g	-	[45]
EDA-PAN	130	-	[46]
PDA@PCL/PEO	-	60.2	[47]
PMMA/zeo	-	95.33	[48]
PVDF@CoAl-LDH	-	621.17	[49]
PAN-g-HPEI	-	194	[50]
PPy@PVDF/PDA	384.6	-	[51]
AOPAN	-	68.07	[52]
PHMG-OCS-PVA	289	-	[53]
MIEX resin	254.58	607.68	[54]
Fe-Mg LDH	894.17	370.36	[55]
CS-A nanofiber pads	1140.70	505.31	[56]
Chitosan/PVA/TiO_2_	131	314	[57]
cLDH	584.56	451.2	[58]
GO-NiFe LDH	450	403	[59]
Copolymergel-NH_2_	-	1281.6	This study
Fe-Copolymergel-NH_2_	3201.9	1089.8	This study

**Table 5:** Thermodynamic parameters of CR and MO adsorption onto Fe- and Copolymergel-NH_2_ at different temperatures.

**Copolymergel-NH_2_**			**ΔG^0^ (kJ/mol)**			
**Dyes**	**ΔH^0^ (kJ/mol)**	**ΔS^0^ (J/Kmol)**	**293 K**	**303 K**	**313 K**	**323 K**
**CR**	15.09	58.78	−2.14	−2.73	−3.32	−3.91
**MO**	−1.5	−3.91	−0.35	−0.31	−0.28	−0.24
**Fe-Copolymergel-NH_2_**			**ΔG^0^ (kJ/mol)**			
**Dyes**	**ΔH^0^ (kJ/mol)**	**ΔS^0^ (J/Kmol)**	**293 K**	**303 K**	**313 K**	**323 K**
**CR**	−34.6	−79.5	−11.29	−10.5	−9.7	−8.91
**MO**	−16.23	−22.67	−9.58	−9.36	−9.13	−8.9

## Data Availability

The original contributions presented in this study are included in the article/Supplementary Materials. Further inquiries can be directed to the corresponding authors.

## Supplementary Materials

The following supporting information can be downloaded at: https://www.mdpi.com/article/10.3390/polym18151838/s1, Figure S1: Swelling degree of Fe- and Copolymergel-NH_2_ in water; Figure S2: Fe-Copolymergel-NH_2_ CR reusability; Table S1: Elemental composition of Fe- and Copolymergel-NH_2_ by XPS analysis; Table S2: SEM-EDS analysis of Fe- and Copolymergel-NH_2_.
