# Supplementary material for: Production of FeCl3-Treated Amine Functional Polymer Gel for Enhanced Removal of Methyl Orange and Congo Red Anionic Dyes
Source: Polymers (Basel). 2026 Jul 27;18(15):1838. doi: 10.3390/polym18151838 (PMC13467684; doi:10.3390/polym18151838)
Supplement: Supplementary file 1 [file polymers-18-01838-s001.zip › polymers-4426955-supplementary.pdf]

# Supporting Information

*Article*

# Production of $FeCl_3$-Treated Amine Functional Polymer Gel for Enhanced Removal of Methyl Orange and Congo Red Anionic Dyes

**Şeyda Getir [1,2,*], Atakan Toprak [1,2,3*] and Baki Hazer [2,3,4]**

[1] Department of Chemistry and Chemical Process Technology, Zonguldak Bülent Ecevit University, 67900 Zonguldak, Turkey
[2] Department of Nano Technology Engineering, Zonguldak Bülent Ecevit University, 67100 Zonguldak, Turkey; baki.hazer@kapadokya.edu.tr

[3] Department of Chemistry, Zonguldak Bülent Ecevit University, 67100 Zonguldak, Turkey

[4] Department of Aircraft Airframe Engine Maintenance, Kapadokya University, 50420 Nevşehir, Turkey
* Correspondence: seyda.getir@beun.edu.tr (Ş.G.); atakantoprak2@hotmail.com or atakantoprak@beun.edu.tr (A.T.); Tel.: +90-3726436201-5128; Fax: +90-3726436604

**Table S1**. Elemental composition of Fe- and Copolymergel-$NH_2$ by XPS analysis

| Element (wt.%) | Copolymergel-$NH_2$ | Fe-Copolymergel-$NH_2$ |
|---|---|---|
| **C 1s** | 67.30 | 56.22 |
| **N 1s** | 2.90 | 3.38 |
| **O 1s** | 22.50 | 27.52 |
| **Cl 2p** | 3.74 | 5.65 |

**Table S2.** SEM-EDS analysis of Fe- and Copolymergel-$NH_2$.

| Element (wt.%) | Copolymergel-$NH_2$ | Fe-Copolymergel-$NH_2$ |
|---|---|---|
| **C** | 58.32 | 56.22 |
| **N** | 6.32 | 6.25 |
| **O** | 33.17 | 30.23 |
| **Cl** | 2.19 | 2.88 |
| **Fe** | - | 4.42 |

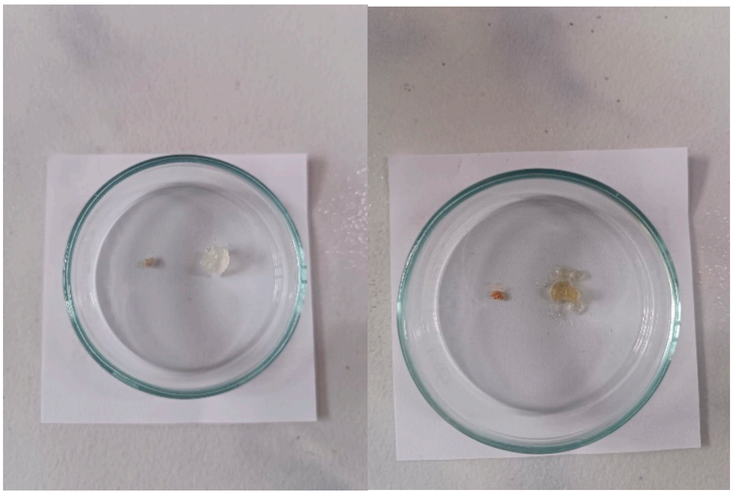

**Fig. S1.** Swelling degree of Fe- and Copolymergel-$NH_2$ in water.

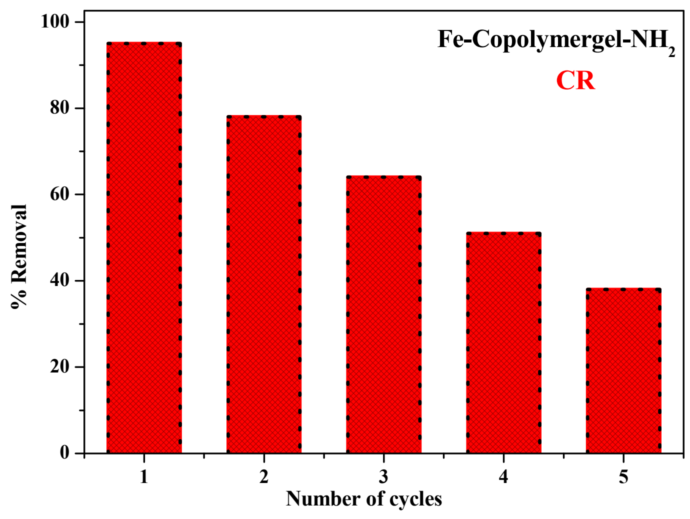


**Figure S2.** Fe-Copolymergel-$NH_2$ CR reusability.
